# Outcomes of the Routine Removal of the Syndesmotic Screw

**DOI:** 10.7759/cureus.26675

**Published:** 2022-07-09

**Authors:** Nzubechukwu Ijezie, Hossam Fraig, Samson Abolaji

**Affiliations:** 1 Surgery, Dorset County Hospital NHS Foundation Trust, Dorchester, GBR; 2 Medicine, All Saints University College of Medicine, Kingstown, VCT

**Keywords:** complication, surgeon preference, weber c, weber b, syndesmotic screw

## Abstract

Background

Ankle joint stabilization with fixation following an injury has been the practice for ankle injuries requiring stabilization. When syndesmotic screws are used to stabilize the ankle joint, the current practice encourages the removal of these screws. However, this study was performed to evaluate the outcomes of patients treated with these screws, with the view to challenging routine screw removal.

Methodology

This was a retrospective study analyzing the records of 52 patients who had been treated with the syndesmotic screw over a two-year span.

Results

Of the 26 patients who did not retain the screw, 84.6% (n = 22) had it removed based on the advice of the surgeon as per the current practice. In total, 19 (73.1%) of these patients had suffered at least one complication over the two procedures. Conversely, of the 23 patients who had retained the screw through one procedure, 14 (60.7%) had at least one complication.

Conclusions

Routine syndesmotic screw removal is associated with increased risks of complications compared to retaining the screws, in addition to not producing a superior outcome for the patients.

## Introduction

The ankle joint, one of the most complex joints in the body, is formed by the distal medial surface of the fibula and the distal surface of the tibia articulating with the talus underneath. This joint is strengthened by syndesmotic ligaments, and as such is described as a syndesmotic joint. This joint is important as it transmits the axial load of the body through the ankle to the foot.

Roughly about 14% of ankle fractures are associated with distal tibiofibular syndesmotic disruption [1]. Following fixation of fractured fragments, additional stability to the joint is achieved by the use of the syndesmotic screw.

No significant difference was noticed in the use of stainless steel and titanium screws [2]. Although stronger resistance to shear stress was provided by the 4.5 mm screw [3], there was no notable biomechanical advantage [4]. Further, it was also believed that the level at which the screw was placed did not have an effect on the outcome [5]. In biomechanical terms, two screws afforded more stability compared to one [6] and can be utilized for more proximal fibular fractures such as Dupuytren and Maisonneuve, as well as neuropathic fractures in diabetics [7]. However, there was no advantage when bioabsorbable and stainless screws were compared [8-10].

Ultimately, the range of motion or outcome is not affected by the position of the foot during screw fixation [11,12]. Post-fixation computerized tomography (CT) imaging in roughly 50% of patients showed that appropriate anatomical reduction was not achieved [13,14], which, in turn, had a knock-on effect on outcomes [15-17].

Traditionally, the screw was removed after three months because it was thought to cause pain during weight-bearing and obstruct the normal ankle function [18,19]. Some have also argued that removal was necessary to attain the final anatomic reduction [20]. Yet, other studies have shown that the removal of the syndesmotic screw does not improve the functional outcome or range of motion [21-25].

One-year post-procedure tibiofibular clear space revealed that there was no significant difference between patients who had had the synsdesmotic screw retained and those who did not [23,25]. However, some authors have provided evidence that tibiofibular diastasis has been noticed following the removal of the syndesmotic screw [26-28].

This study aimed to evaluate the outcomes of patients treated with syndesmotic screws, with an emphasis on the complications experienced by patients and to challenge the need for routine syndesmotic screw removal.

## Materials and methods

This study was conducted by retrospectively reviewing the records of patients who had undergone treatment for ankle injuries between 2019 and 2020 at Dorset County Hospital. Patients included in the study involved those who had been treated with syndesmotic screws for their ankle injuries.

The retrospective design was adopted to illustrate clearly which practice was deemed appropriate, as some of these patients were reviewed at a later date in the clinic. The patient population comprised those who had (1) Weber B and (2) Weber C fractures. Patients who were treated with button-type ankle fixation were excluded.

Data for the study were gathered by reviewing data and electronic records for patients who underwent surgical treatment for ankle injuries with screw fixation over a period of two years to compare their outcomes.

## Results

In total, 52 patients, comprising 21 males and 31 females, over a period of two years between 2019 and 2020 were managed with screws for injuries related to the ankle joint. Inclusion for this study was based on patients who had undergone ankle reduction and treatment that utilized the syndesmotic screw. One patient was excluded from the study as they were treated with suture button fixation following their ankle injuries.

Patients were aged between 20 and 86. Of all patients treated, 31 (59.6%) were reduced with one screw, another 20 (38.4%) were reduced with two screws, and one (1.9%) was reduced with three screws.

Ankle injury classifications reduced with the syndesmotic screw comprised Weber type B (n = 35; 67.3%) and Weber type C (n = 17; 32.7%) fracture classifications.

Overall, 26 of 52 patients had a subsequent procedure to have their syndesmotic screws removed while 23 patients retained the syndesmotic screw. There was no documentation regarding the outcome for three other patients.

**Figure 1:**
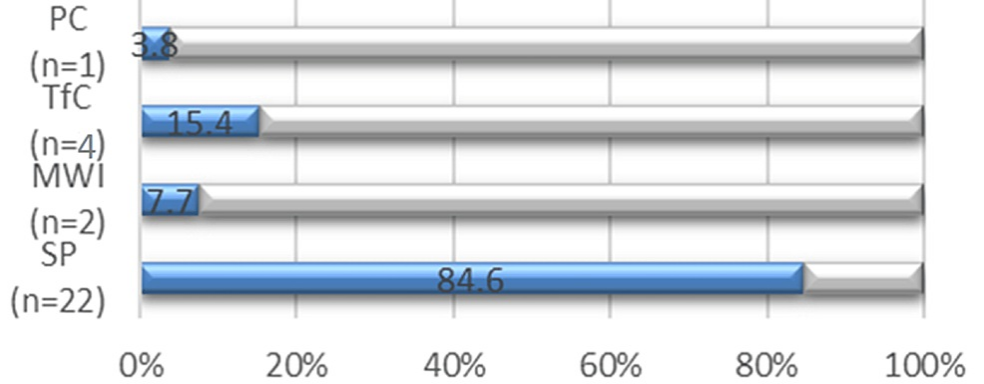
Documented reason for screw removal. SP: surgeon’s preference; MWI: metalwork issues; TfC: treatment for complications; PC: patient’s choice

As illustrated in Figure 1, most patients (n = 22; 84.6%) had had their syndesmotic screw removed due to the preference of the surgeon, which could be implied explicitly by informing the patient that the screw had to come off later (probably mirroring current practice) or implicitly by persuading the patient to conform to current treatment practice as a means to a better outcome. Four (15.4%) patients had to undergo a subsequent syndesmotic screw surgery based on their presenting complications, whereas one (3.8%) patient was documented to have the syndesmotic screw removed by choice. Further, two (7.7%) had to have a subsequent procedure due to metalwork issues.

**Figure 2:**
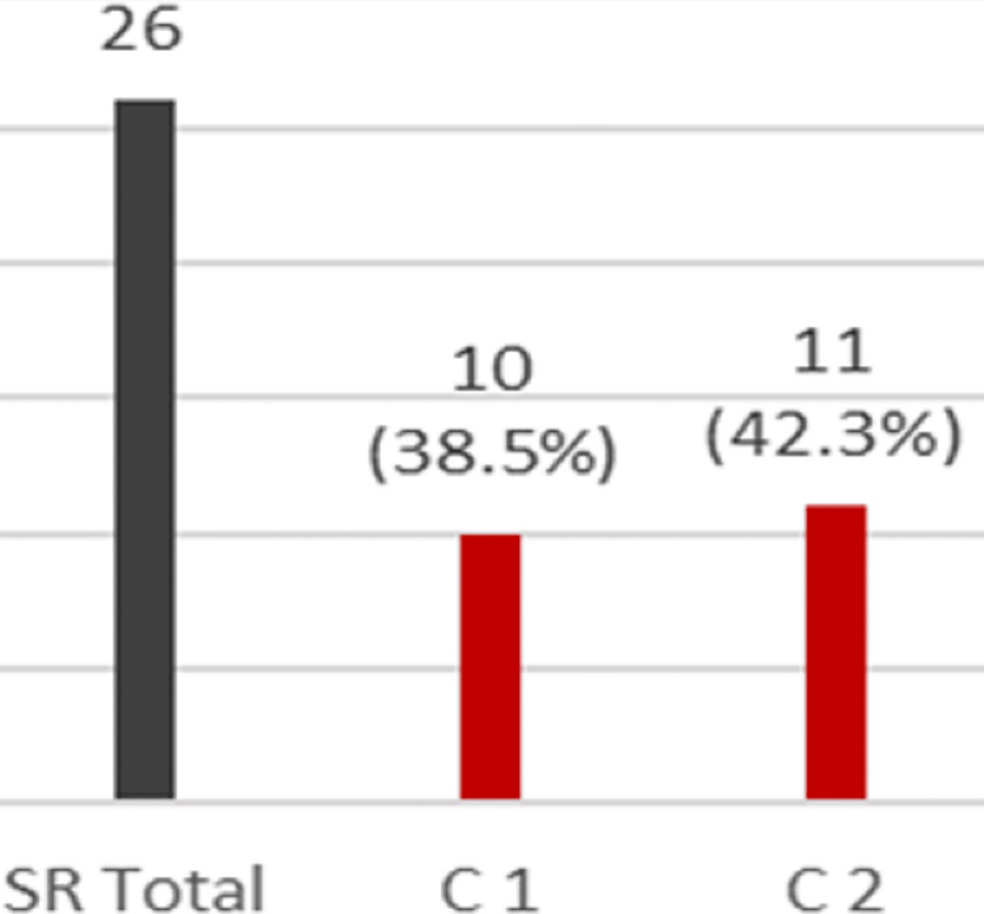
Complications following initial and subsequent procedures. SR Total: total number of patients who did not have the screw retained; C 1: number of patients who had complications following screw fixation; C 2: number of patients who had complications following screw removal

Collated data revealed that of the 26 patients who had had the syndesmotic screw removed, 19 (73.1%) suffered at least one complication from either the initial procedure, the subsequent procedure, or both. Figure 2 depicts the number of patients who experienced at least one complication following the initial and/or subsequent procedure for patients who had the syndesmotic screw removed with 10 (38.5%) and 11 (42.3%), respectively.

**Figure 3:**
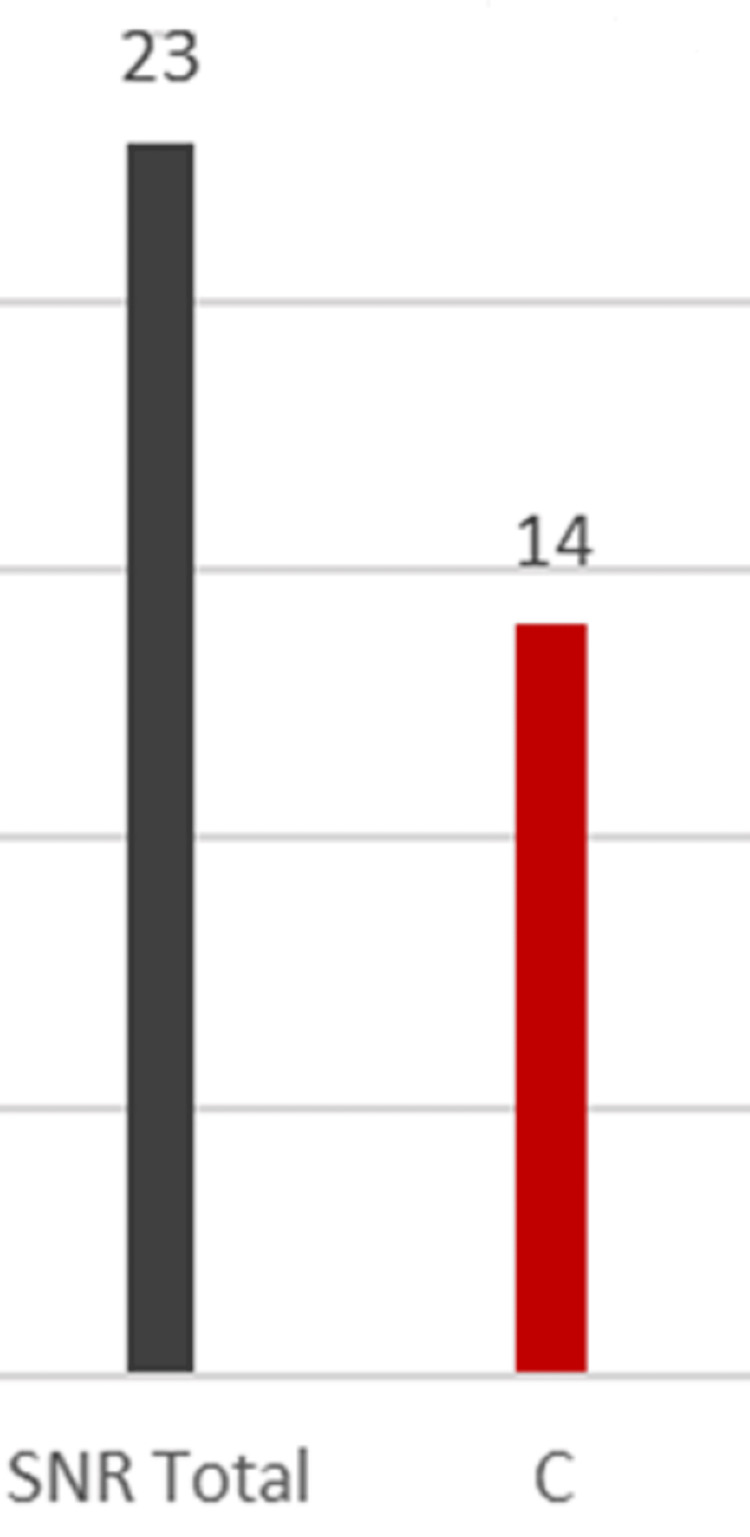
Complications for patients who had the screw retained. SNR: total number of patients who had syndesmotic screw retained; C: number of patients who had complications following screw retention

As illustrated in Figure 3, 14 (60.7%) of 23 patients had experienced at least one complication.

From the data analyzed, it was evident that most of these complications could have been avoided as many of the screws were removed due to what was recommended to the patients.

Nevertheless, there were complications that were observed to be peculiar to patients who did not have the screw retained. Some of these complications, following removal of the syndesmotic screw, were wound dehiscence (7.7%), limp (3.8%), wound ulcer (7.7%), and clicking within the joint (3.8%). Further, other rare but significant complications noted in the wake of screw removal were syndesmotic joint widening (19.2%), pain over the medial aspect of the ankle post-screw removal (3.8%), feeling of instability/loose body around the ankle joint (11.5%), superficial peroneal nerve weakness, and weak inversion/plantar flexion (11.5%).

In contrast, patients who had the syndesmotic screw retained experienced mottling (8.7%), antalgic gait (4.3%), and forward subluxating equinus (8.7%), in addition to the commonly observed complications.

Overall, 34.6% of patients who did not have the syndesmotic screw retained suffered minimal or no complications. However, 15.4% of these patients had broken the screw between four and six months after the first procedure, whereas 56.5% of patients who had the syndesmotic screw retained experienced minimal or no complications without any documented effect on the eventual patient outcome.

From the above, it is clearly evident that there were more complications experienced by patients who did not have the syndesmotic screw retained. Further, some of the patients who had retained the screws did so due to the complications they suffered after the fixation procedure, further buttressing the point that routine screw removal is not necessary but should only be carried out when there are clear indications for it as it carries more risks of undesirable outcomes and complications.

## Discussion

The study revealed that routine removal of the syndesmotic screw carried a higher risk of complications at 42.3% compared to the 39% of patients who had had the syndesmotic screw retained.

In considering complications one must bear in mind that inherent in all surgical procedures is some degree of complications. The fixation of the ankle with the syndesmotic screws is no different as over the course of this study some complications were noted to be common following the procedure irrespective of whether the syndesmotic screw was retained. Some of these include stiffness, joint ache and pain, swelling, muscle wasting, tight calf, and chronic regional pain syndrome. Others included wound breakdown and infection.

After following 30 patients for a period of 36 months, De Souza et al. [29] demonstrated that it was more beneficial to have the syndesmotic screw retained.

There are studies showing that the removal of the syndesmotic screw does not improve the functional outcome or range of motion [21-25]. Removal of the syndesmotic screw is complicated by surgical site infection (SSI) in up to 9% of cases [25]. Because of the high complication rate, it would be beneficial to remove the screw only if the patient experienced difficulties.

Pogliacomi et al. [23] demonstrated it was not prudent to not retain the syndesmotic screw, citing an increased risk to patients of syndesmotic diastasis, even as Kaftandziev et al. [24] opined that the patients who retained the syndesmotic screw fared better, despite the screw being broken. Further, a similar conclusion was arrived at by Manjoo et al. [22] who observed that broken screws did not result in an adverse effect on outcomes as broken screws had similar or improved outcomes over intact screws.

This study was limited by the number of patients included in the study. Further, a multicenter study would be advocated, in addition to a study design that actively follows the patients and monitors changes in symptoms and clinical presentations. A prospective randomized trial would be invaluable in evaluating the outcome for both groups of patients.

## Conclusions

This study revealed a higher complication rate for routine screw removal compared to screw retention. In fact, the rate of complications was noticed to be much lower in the group of patients that had had the syndesmotic screw retained.

To this end, routine screw removal as the standard of practice needs to be discouraged. This would eliminate the added burden of the suffering patients have to experience when injuries requiring fixation of the ankle occur as there was no evidence of superior outcomes from non-retention of the screw. Overall, the gains from this method of practice would be astronomical not only in clinical terms but also in economic terms.

However, this study was limited by patient size and the fact that it was a non-randomized study. As stated in the discussion, a multicenter study adopting the double-blind approach would be useful in driving home the fact that routine syndesmotic screw removal is a practice that should cease.

## References

[1] Dattani R, Patnaik S, Kantak A, Srikanth B, Selvan TP (2008). Injuries to the tibiofibular syndesmosis. J Bone Joint Surg Br.

[2] Beumer A, Valstar ER, Garling EH, Niesing R, Heijboer RP, Ranstam J, Swierstra BA (2005). Kinematics before and after reconstruction of the anterior syndesmosis of the ankle: a prospective radiostereometric and clinical study in 5 patients. Acta Orthop.

[3] Hansen M, Le L, Wertheimer S, Meyer E, Haut R (2006). Syndesmosis fixation: analysis of shear stress via axial load on 3.5-mm and 4.5-mm quadricortical syndesmotic screws. J Foot Ankle Surg.

[4] Thompson MC, Gesink DS (2000). Biomechanical comparison of syndesmosis fixation with 3.5- and 4.5-millimeter stainless steel screws. Foot Ankle Int.

[5] Kukreti S, Faraj A, Miles JN (2005). Does position of syndesmotic screw affect functional and radiological outcome in ankle fractures?. Injury.

[6] Xenos JS, Hopkinson WJ, Mulligan ME, Olson EJ, Popovic NA (1995). The tibiofibular syndesmosis. Evaluation of the ligamentous structures, methods of fixation, and radiographic assessment. J Bone Joint Surg Am.

[7] Thordarson DB (2004). Ankle fractures in diabetics. Techn Foot Ankle Surg.

[8] Kaukonen JP, Lamberg T, Korkala O, Pajarinen J (2005). Fixation of syndesmotic ruptures in 38 patients with a malleolar fracture: a randomized study comparing a metallic and a bioabsorbable screw. J Orthop Trauma.

[9] Sinisaari IP, Lüthje PM, Mikkonen RH (2002). Ruptured tibio-fibular syndesmosis: comparison study of metallic to bioabsorbable fixation. Foot Ankle Int.

[10] Thordarson DB, Samuelson M, Shepherd LE, Merkle PF, Lee J (2001). Bioabsorbable versus stainless steel screw fixation of the syndesmosis in pronation-lateral rotation ankle fractures: a prospective randomized trial. Foot Ankle Int.

[11] Bragonzoni L, Russo A, Girolami M, Albisinni U, Visani A, Mazzotti N, Marcacci M (2006). The distal tibiofibular syndesmosis during passive foot flexion. RSA-based study on intact, ligament injured and screw fixed cadaver specimens. Arch Orthop Trauma Surg.

[12] Olerud C (1985). The effect of the syndesmotic screw on the extension capacity of the ankle joint. Arch Orthop Trauma Surg (1978).

[13] Gardner MJ, Demetrakopoulos D, Briggs SM, Helfet DL, Lorich DG (2006). Malreduction of the tibiofibular syndesmosis in ankle fractures. Foot Ankle Int.

[14] Schwarz N, Kofer E (2005). Postoperative computed tomography-based control of syndesmotic screws. Eur J Trauma.

[15] Egol KA, Pahk B, Walsh M, Tejwani NC, Davidovitch RI, Koval KJ (2010). Outcome after unstable ankle fracture: effect of syndesmotic stabilization. J Orthop Trauma.

[16] Leeds HC, Ehrlich MG (1984). Instability of the distal tibiofibular syndesmosis after bimalleolar and trimalleolar ankle fractures. J Bone Joint Surg Am.

[17] Weening B, Bhandari M (2005). Predictors of functional outcome following transsyndesmotic screw fixation of ankle fractures. J Orthop Trauma.

[18] Bell DP, Wong MK (2006). Syndesmotic screw fixation in Weber C ankle injuries--should the screw be removed before weight bearing?. Injury.

[19] Miller AN, Paul O, Boraiah S, Parker RJ, Helfet DL, Lorich DG (2010). Functional outcomes after syndesmotic screw fixation and removal. J Orthop Trauma.

[20] Amouzadeh Omrani F, Kazemian G, Salimi S (2019). Evaluation of syndesmosis reduction after removal syndesmosis screw in ankle fracture with syndesmosis injury. Adv Biomed Res.

[21] Briceno J, Wusu T, Kaiser P, Cronin P, Leblanc A, Miller C, Kwon JY (2019). Effect of syndesmotic implant removal on dorsiflexion. Foot Ankle Int.

[22] Manjoo A, Sanders DW, Tieszer C, MacLeod MD (2010). Functional and radiographic results of patients with syndesmotic screw fixation: implications for screw removal. J Orthop Trauma.

[23] Francesco P, Carlotta A, Sara R, Filippo C, Enrico V, Francesco C (2019). The management of syndesmotic screw in ankle fractures. Acta Biomed.

[24] Kaftandziev I, Spasov M, Trpeski S, Zafirova-Ivanovska B, Bakota B (2015). Fate of the syndesmotic screw--search for a prudent solution. Injury.

[25] Boyle MJ, Gao R, Frampton CM, Coleman B (2014). Removal of the syndesmotic screw after the surgical treatment of a fracture of the ankle in adult patients does not affect one-year outcomes: a randomised controlled trial. Bone Joint J.

[26] Jordan TH, Talarico RH, Schuberth JM (2011). The radiographic fate of the syndesmosis after trans-syndesmotic screw removal in displaced ankle fractures. J Foot Ankle Surg.

[27] Hsu YT, Wu CC, Lee WC, Fan KF, Tseng IC, Lee PC (2011). Surgical treatment of syndesmotic diastasis: emphasis on effect of syndesmotic screw on ankle function. Int Orthop.

[28] Song DJ, Lanzi JT, Groth AT, Drake M, Orchowski JR, Shaha SH, Lindell KK (2014). The effect of syndesmosis screw removal on the reduction of the distal tibiofibular joint: a prospective radiographic study. Foot Ankle Int.

[29] de Souza LJ, Gustilo RB, Meyer TJ (1985). Results of operative treatment of displaced external rotation-abduction fractures of the ankle. J Bone Joint Surg Am.

